# Implementing shared decision-making: consider all the consequences

**DOI:** 10.1186/s13012-016-0480-9

**Published:** 2016-08-08

**Authors:** Glyn Elwyn, Dominick L. Frosch, Sarah Kobrin

**Affiliations:** 1 The Dartmouth Institute for Health Policy and Clinical Practice, 37 Dewey Field Road, Hanover, NH 03755 USA; 2 Palo Alto Medical Foundation Research Institute, 795 El Camino Real, Palo Alto, CA 94301 USA; 3 Department of Medicine, University of California, Los Angeles, CA 90024 USA; 4 National Cancer Institute, 9609 Medical Center Drive, Rockville, MD 20850 USA

**Keywords:** Collaborative deliberation, Shared decision making, Patient-centered care, Implementation, Practice improvement, Quality improvement, Multilevel, Conceptual model, Measurement

## Abstract

**Background:**

The ethical argument that shared decision-making is “the right” thing to do, however laudable, is unlikely to change how healthcare is organized, just as evidence alone will be an insufficient factor: practice change is governed by factors such as cost, profit margin, quality, and efficiency. It is helpful, therefore, when evaluating new approaches such as shared decision-making to conceptualize potential consequences in a way that is broad, long-term, and as relevant as possible to multiple stakeholders. Yet, so far, evaluation metrics for shared decision-making have been mostly focused on short-term outcomes, such as cognitive or affective consequences in patients. The goal of this article is to hypothesize a wider set of consequences, that apply over an extended time horizon, and include outcomes at interactional, team, organizational and system levels, and to call for future research to study these possible consequences.

**Main argument:**

To date, many more studies have evaluated patient decision aids rather than other approaches to shared decision-making, and the outcomes measured have typically been focused on short-term cognitive and affective outcomes, for example knowledge and decisional conflict. From a clinicians perspective, the shared decision-making process could be viewed as either intrinsically rewarding and protective, or burdensome and impractical, yet studies have not focused on the impact on professionals, either positive or negative. At interactional levels, group, team, and microsystem, the potential long-term consequences could include the development of a culture where deliberation and collaboration are regarded as guiding principles, where patients are coached to assess the value of interventions, to trade-off benefits versus harms, and assess their burdens—in short, to new social norms in the clinical workplace. At organizational levels, consistent shared decision-making might boost patient experience evaluations and lead to fewer complaints and legal challenges. In the long-term, shared decision-making might lead to changes in resource utilization, perhaps to reductions in cost, and to modification of workforce composition. Despite the gradual shift to value-based payment, some organizations, motivated by continued income derived from achieving high volumes of procedures and contacts, will see this as a negative consequence.

**Conclusion:**

We suggest that a broader conceptualization and measurement of shared decision-making would provide a more substantive evidence base to guide implementation. We outline a framework which illustrates a hypothesized set of proximal, distal, and distant consequences that might occur if collaboration and deliberation could be achieved routinely, proposing that well-informed preference-based patient decisions might lead to safer, more cost-effective healthcare, which in turn might result in reduced utilization rates and improved health outcomes.

## Background

Shared decision-making is widely considered good in and of itself [1]: healthcare professionals have a duty to inform people about the benefits and harms of proposed interventions. These duties are at the core of good clinical practice. Shared decision-making is defined by extending this duty to supporting people to arrive at informed preferences, eliciting and respecting those preferences by integrating them as decisions are made [2]. Shared decision-making is distinguished from various models of patient-clinician communication, such as motivational interviewing, by being focused on choice rather than change [3]. The process is described in more detail using the terms collaborative deliberation [4]. We use the terms shared decision-making and collaborative deliberation interchangeably in this article. Shared decision-making does not advocate deliberation in every interaction or for every decision—that would be impractical. Rather, a deliberative approach is suggested where the existence of reasonable alternatives justify the work of informing patients so that they are able to form and share preferences about them.

It has been argued that shared decision-making represents the pinnacle of patient-centered care [5]. Epstein’s summary of the evidence about the healing effect of good communication [6] has reinforced the ethical imperative that practice should be based on collaboration and deliberation. Yet, despite these arguments and this evidence, shared decision-making is not widely adopted in routine clinical practice [7, 8].

Powerful influences drive the practice of medicine, and so an ethics-based, *deontological* view, alone, does not lead to significant change in how healthcare is organized and delivered. Researchers respond by generating evidence to support a *consequentialist* viewpoint. Studies are typically designed to investigate how shared decision-making might improve short-term outcomes that are relevant to both patients and others. Accepting that neither ethical arguments, nor rational evidence, alone or in combination, are the only factors that predict change in clinical practice, we noticed limitations in how researchers have evaluated shared decision-making in published studies.

Evaluation metrics for shared decision-making have been focused on short-term outcomes, mostly assessing cognitive or affective effects on patients, with limited agreement about how to value, and in some cases even how to measure the desired outcomes. These short-term outcomes have consequences in the longer term, of course, leading to calls for healthcare delivery researchers to consider broader contexts over time, including interventions that simultaneously consider patients, clinical teams, organizations and systems in which they deliver care. These calls suggest the need to go beyond the evaluation of dyadic interactions to “include examination of mechanisms and measure of the effectiveness of healthcare organizations and health systems in improving outcomes” [9]. Future evaluations might have more impact if they were able to measure consequences that are valued by a wider array of stakeholders: patients, clinicians, as well as organizational managers and executives, and policy makers [10].

At a time when the sustainability of healthcare delivery systems is questioned because of high costs and overzealous medicalization, it is possible that shared decision making might be an approach that gains wider acceptance. But existing efforts to study shared decision-making have mostly focused on how clinicians interact with patients, without paying attention to team, organizational, and system level factors in which these interactions are embedded. Existing work therefore has for the most part not provided evidence of sustained change in practice, nor have the tools that have been advocated become widely embedded into delivery systems [10, 11]. Despite the policy interest in shared decision-making, the majority of organizations and delivery systems of care delivery cite barriers and remain indifferent to whether shared decision-making is taking place, with or without patient decision aids [12].

Reflecting on the existing research on shared decision-making, we suggest that the work to date has had too narrow a focus, with insufficient agreement on the conceptualization of shared decision-making, as well as a conflation of two topics, a shared decision-making process and the use of patient decision aids.

Most definitions of shared decision-making call for high-quality information and the participation of patients in a process of decision-making. That much is uncontroversial. However, debate continues on several key issues that affect assessment of the quality of a shared decision-making process and how to value commonly measured short-term outcomes, e.g., the ideal *role* of patients in a decision-making process and *decision conflict* as an outcome, and whether current measures accurately capture the increased ambivalence that arises when options are recognized. Should the preferred role of a patient always be followed (rather than compel patients to engage in all decisions)? Insisting that patients engage in decision-making is the position described by the term *mandatory autonomy* [13]. This approach does not receive wide support but some ethicists argue for it. Most advocates of shared decision-making take the view that patients should be engaged in decisions to the extent that they wish to do so—a position described by the term *optional autonomy* [13], as there is some evidence that doing so is helpful [14]. Nevertheless, further research in this area is needed to clarify how patient participation, whether desired or not, affects health outcomes across settings. In terms of cognitive-affective outcomes, clarity about the ideal psychological pathway—from option recognition, to meaningful comparison, and the resolution of the inherent emotional work of choosing among alternatives, has not yet been fully elucidated. Clarity about expected proximal measures could help us. Different conceptualizations, measured differently, produce results that are difficult to compare [15]. Meaningful debate of these and other questions requires competing hypotheses to be tested.

A second problem, and one which has been partly responsible for the narrow focus, is the *conflation* of a shared decision-making process and the use of patient decision aids. For a decade or more, there seemed to be a belief that tools designed to deliver *information* to patients could lead patients and clinicians to engage in a process of collaboration and deliberation. This belief was so prevalent that only one of the randomized trials of patient decision support conducted between 1990 and 2006 made an effort to study the *interactional* process in clinical encounters [16]. In other studies of patient decision aids, patients *reported* being more involved in decisions but there was no confirmatory evidence from observational data. This changed when a research group at the Mayo Clinic began to use *observer-based* measures as well as patient-reported outcomes [17]. It is worth noting that the tools under investigation in the Mayo trials were specifically designed to create collaborative conversations [18], so the data are not strictly comparable to studies of patient decision aids given to patients before clinical encounters. This conflation has delayed progress on research into the process of shared decision-making, viewed as a two-way communication. It has rather led to an emphasis on patient decision aids, to their development, evaluation, and to the development of quality “standards.” Insufficient work has been done testing the assumption that the use of these tools would lead to shared decision-making [19]. At the same time, this conflation led to significant policy emphasis and legislative change. The Affordable Care Act in the USA [20] supported shared decision-making; however, the wording of the law implies that it could be achieved by incentivizing the use of patient decision aids [21], and the development of a certification process to standardize the quality of these tools, when in fact, we have no evidence that tools on their own will achieve this goal.

Nevertheless, the results of over 100 trials of patient decision aids have been consistent. After exposure to the tools, patients’ knowledge increases, patients make more accurate assessments of risk, they *report* more involvement in decision-making, they do not become more anxious, and they report being more satisfied and more confident in their decisions [22]. These, in broad terms, are the typical outcomes that have been measured—described by Shay and Lafata as cognitive-affective outcomes [23]. These outcomes are typically measured soon after the relevant clinical interactions, testing the hypothesis that exposure to patient decision aids leads to positive effects on knowledge, satisfaction, and perceived involvement, without increasing regret or decisional conflict.

We suggest that this *conceptualization* of shared decision-making is too narrow; it fails to address the potential relationship between shared decision-making and a range of consequences that might occur over a longer span of time if healthcare professionals embraced the tasks of collaborating with patients to carefully consider the harms and benefits of potential alternatives.

We argue that it would be timely to consider a wider range of outcomes where collaborative deliberation has been achieved [4]. To do so, we would assume that the research would be able to study settings where clinicians were both able and willing to accomplish shared decision-making or that both patients and clinicians were able to use patient decision support tools as required, or both. Such settings are difficult to achieve, given that space, resources, and context will be effect modifiers. Current healthcare settings, in the USA and elsewhere, have financial and other incentives that are not well-aligned to support collaboration and deliberation with patients about alternative options, although these are changing with growing emphasis on value-based payment methods [24]. We recognize of course that achieving ideal conditions for shared decision-making to have its greatest impact is not easy, but for the purposes of developing a hypothetical framework—such an assumption allows us to focus on *consequences* rather than worry here about the fidelity to shared decision-making.

The aim of this article is to analyze the work done in this area and to propose a conceptual framework that hypothesizes the impact of collaborative deliberation at individual, interactional, organization, and system levels. We describe opportunities to examine these consequences and the possibility of evaluating causal pathways.

## Approach

To achieve our goals, we took the following approaches:

1. We considered published work that has examined outcomes relating to shared decision-making. We focused on systematic reviews and other high-quality narrative reviews.
2. We identified research gaps highlighted by existing reviews and discussed the preliminary results of our work at academic meetings and conferences.
3. We developed a multilevel framework to describe potential consequences over time and propose a sample of questions to illustrate potential future research directions.

To do the work, we examined a review of outcomes considered in existing studies of shared decision-making [23] and analyzed the contribution of patient decision aids [22]. In addition, we reviewed other relevant systematic reviews, including an assessment of whether patient decision support had led to cost savings [25], the effect of shared decision-making on health inequalities [26], on litigation [27], and on treatment satisfaction and clinical outcomes [28]. After reading these reviews, we met, face-to-face and virtually, on multiple occasions. Between meetings, we used a cloud-based document editing system to develop a figure, tables, and manuscript. A draft version of the framework was submitted to a relevant conference [29], where feedback shaped a penultimate version. We imagined a range of possible consequences, considering they might be good for some but not for others, recognizing that different consequences might not be consistently valued by different stakeholders. As we developed the framework, we decided to group outcomes into those that were *proximal*, *distal*, and *distant* to a shared decision-making process.

**Fig. 1:**
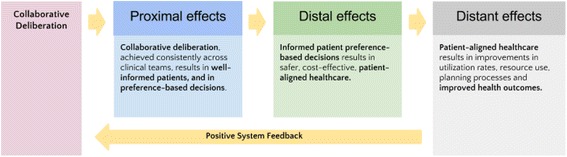
The potential consequences of collaborative deliberation

We categorized outcomes by thinking about the *level of impact*. Figure 1 provides an overview of a process where collaborative deliberation leads to an series of hypothesized effects. Table 1 provides more detail. We did not wish to impose definitive time limits because we acknowledge that divisions will not be precise, and some consequences might accrue gradually [11]. Parsing potential outcomes into these time frames enabled us to consider the consequences of collaborative deliberation that go beyond those that are obvious or easily measured.

**Table 1:** The potential multilevel consequences of collaborative deliberation

Accomplishing collaborative deliberation	Proximal effects	Distal effects	Distant effects
	Informed preference; cognitive and affective effects	Cautious decisions; modified relationships; enduring trends	Modified utilization and resource use; modified help seeking behavior
Individual levels	Preferences for outcomes and treatments based on comprehension of high-quality evidence.	Collaborative deliberation generates different clinician-patient and patient-care team relationships—with positive and negative potential.	Increased use of interventions that lower risk of harms, raise likelihood of benefits.
			Lower utilization of high-risk, marginal-benefit interventions.
	Potential conflict where informed patient preference is not supported by clinician or organizational policies.		
		Improved adherence to selected options and less regret about choices made, improved resilience and self-efficacy.	
			Greater engagement in assessing the long-term value of interventions, leading to lower service utilization and improved self-management by patients.
	Realistic expectations with possible changes in confidence and satisfaction levels.		
		Clinicians experience the synergy of working to aligned organizational-based incentives.	
			Potential for reduced risk of professional burnout.
	Reduced intention to choose intensive treatment in some settings.		
	Clinicians experience intrinsic reward for work done well.		
	Clinicians experience the cognitive and emotional work of supporting patients making decisions.		
Interactional and group levels	Enhanced relationships with clinicians and with clinical teams	Development of team culture that generates realistic expectations and judicious use of resources	Norms established: collaboration and deliberation become expected behaviors
	Dissatisfaction due to decisional burden, decisional conflict, uncertainty, and concern about honoring patient preferences.		
		Patients prompted to ask questions, assess the value of interventions	
		Enhanced relationships reduce complaints and legal challenges.	
	Exhibiting respect for individuals’ informed preferences leads to increased satisfaction with care, at dyadic and group levels.		
Organizational levels	Many clinicians in an organization become willing to share information, with patients, about alternative options.	Higher aggregate patient experience scores, e.g., satisfaction with care	Change in resource utilization requires workforce changes.
			Different utilization patterns lead to changes in delivery infrastructure and capacity.
		Higher scores on organizational measures of patient-centered care.	
	Organizational commitment to resource, promote, and sustain collaborative deliberation.		
		Fewer legal challenges.	
		Improved staff morale, lower incidence of professional burnout, and less absenteeism.	
	Redesign of workflow, space, and information systems will short term require investment.		
Healthcare system level	Collaborative deliberation viewed as normative, therefore embedded in policies, systems, and rewards.	Greater skepticism and scrutiny of new drugs, interventions, and services.	Lower resource utilization, with trends to more cost-effective care, that leads to changes in strategic investment decisions.
		Improved cost-effectiveness.	System level interest population health, and its determinants.
	Recruits learn and follow different policies.		
		Reduction in malpractice costs.	

## Main argument

Many measurement approaches to shared decision-making have been developed [22, 30, 31]. Shay and Lafata noted that “the empirical evidence” about the effectiveness of shared decision-making has not been “systematically summarized” and set about the task [23]. Shay and Lafata found three sets of reported measures: (1) cognitive-affective, (2) behavioral, and (3) health outcomes, reporting that patient “health outcomes were the least studied.” There were no efforts made to suggest other types of consequences.

Significant efforts to implement patient decision aids have taken place, mainly in the USA and in the UK [32]. The hospital system at Dartmouth Hitchcock was the first center dedicated to delivering patient decision aids [32], and more than a decade later, it continues to offer such tools to some categories of patients. More recently, Group Health in Seattle implemented a number of patient decision tools alongside a quality improvement initiative aimed at surgical specialists. Evaluations at Group Health demonstrated high levels of patient exposure to the tools, higher than those achieved in other organizations, and evidence of impact on surgical rates [33]. Others have reported difficulties in attempting to introduce these tools into organizations [32, 34]. Reviews of efforts made to implement patient decision aids indicate how difficult it is to alter routine workflows [35]. Most of the research however has focused on how to provide decision aids to specific patient groups before they attend clinic or on how to motivate clinicians to provide decision aids to relevant patient groups. Much less attention has been given to research that would identify organizational, system, and policy factors that could influence practice change.

Few of these implementation-type studies have considered the effects of shared decision-making, beyond the proximal outcomes of decision support interventions given to patients. The work at Group Health is a notable exception [28, 33]; their positive results serve to illustrate what might be possible. Even so, they did not evaluate a fuller range of consequences or over a time horizon. It is not therefore possible to conclude much at all about the mid or longer-term consequences of a concerted effort to undertake shared decision-making at a system wide level, whether defined as decision aid use or as a communicative process of collaboration and deliberation. A handful studies have included costs and cost-effectiveness, but these have all been related to the evaluation of patient decision aids [25]. The results of a systematic review of studies with economic assessments revealed the need for more and higher quality studies before firmer conclusions could be drawn [25]. A systematic review of shared decision-making in relation to litigation found five studies, mostly case reports, and was unable to reach a firm conclusion [27].

### Conceptual framework

In this section, we provide a figure which illustrates a hypothesized set of proximal, distal, and distant consequences of collaboration and deliberation undertaken routinely (see Fig. 1), proposing that well-informed preference-based patient decisions may lead to safer, cost-effective healthcare, which in turn reduce utilization rates and improve health outcomes.

To illustrate the potential positive, negative, and potentially peverse consequences, we provide more detail (see Table 1), where we describe a range of potential consequences, with commentary below.

#### Individual consequences—patient and clinician level

As Shay et al. noted, outcomes at the individual level have been those most commonly considered, where the majority of interventions have been decision aids given to patients before clinical encounters and the effects evaluated soon afterwards [23]. It is reassuring to see that such tools show that patients develop preferences based on their comprehension of accessible information, have more realistic expectations, less “decisional conflict” as currently measured, and greater satisfaction. Emerging evidence also suggests that shared decision-making can improve adherence and increase trust [8, 36, 37]. It is also reassuring that these tools do not cause significant anxiety or other problems for patients.

That these tools often reduce the intention to choose intensive treatments has led to most speculation about the prospect of modified resource use and possible cost reduction at distal and distant time points. Nevertheless, this outcome is understudied [25]. Because of the need for long-term follow-up, studies that use modeling methods and sensitivity analyses might be helpful. We know of no qualitative studies that investigated the reasons for the lowered interest in some treatments.

Individual level effects on clinicians should also be considered; the experience of supporting patients in arriving at informed decisions may be intrinsically rewarding. Clinicians may also find the effort involved emotionally and cognitively taxing, adding to their workload burden. These consequences for clinicians should be evaluated and understood as they should be expected to influence the uptake of shared decision-making.

#### Interactional and group level consequences

Few studies have examined the impact of shared decision-making as a communicative process beyond the effect on patients. Experts in organizational psychology consider five types of potential outcomes: (1) tangible outcomes products of teaming, e.g., costs or rates; (2) attitudes or emergent states, e.g., trust, psychological safety; (3) cognitive states, e.g., shared mental models; (4) team behaviors, e.g., turnover or absenteeism; and (5) norms, e.g., expected behaviors [38]. As the table proposes, effective shared decision-making may lead to positive effects at interactional levels, by which we mean, to enhanced relationships between patients and clinicians, and to positive communication and processes in clinical teams and clinical microsystems [39, 40]. Demonstrations that professionals are willing to inform and then respect patient preferences might lead to significant positive changes in patient experience, including satisfaction metrics, although there remains the risk that some patients will react negatively to being asked to participate in decisions and become more aware of uncertainty about treatment effects. Clinical encounters that become more difficult to conduct because patients might not want to take responsibility for participating in decisions could lead to clinicians who avoid this approach—such a consequence would need to be monitored. Longer term interactional consequences of adopting shared decision-making might be the development of a culture where careful deliberation and caution are regarded as guiding principles, where patients are coached to assess the long-term value of interventions, to question the benefits and assess their burdens [4]. Given the evidence that teamwork failures correlate strongly with technical clinical errors [41], sustained collaborative deliberation with patients across teams might lead to social norms that become self-sustaining, which could be measured. Resource utilization and other performance scores may improve at group levels [42].

#### Organizational level consequences

We view a healthcare organization as one composed of multiple delivery teams or microsystems and of a size that needs management by higher order functions such as finance, human resources, and so on, led by an executive group. For an organization to accomplish shared decision-making consistently, many clinicians will need to be willing to share information with patients about alternative options with patients to support the comparisons and consider patient preferences. These tasks will need knowledge, skills, and, perhaps most importantly, a widespread shift in attitudes. As we have already noted, patient decision tools help these processes, especially if integrated into workflows and electronic record systems. However, negative consequences will also surface: organizations might resist the investment required and decline to redesign workflow to facilitate the use of patient knowledge tools or refuse to redistribute or substitute work roles. The professional shifts needed to adopt the attitudes and skills required might be too risky for organizations and could lead to workforce problems, given that the work required to confirm that shared decision-making was being provided would modify existing work patterns.

Again, achieving high-quality shared decision-making would likely lead to different scores on measures of patient experience of care which are of increasing importance as organizational benchmarks. This is especially true if the next generation of patient experience measures focus on care that has been tailored to individual preferences. Arguably, such a process could lead to positive consequences, such as fewer complaints and fewer legal challenges. Anecdotally, many organizations have invested in patient information provision, including patient decision aids, because they are convinced that well-informed patients are less likely to conduct lawsuits (personal communication, Emmi Solutions). Expecting and supporting clinicians to achieve shared decision-making could also have a positive effect on organizational morale and could over time improve lower the incidence of professional burnout by better aligning clinician’s values with the work they do [43]. In the long term, if shared decision-making does lead to substantial changes in resource utilization—perhaps reductions in use and cost—clinical workforce composition will have to be modified. Despite the gradual shift toward value-based payment for services, some organizations, motivated by continued income derived from achieving high volumes of procedures and contacts, will see this consequence as negative. The need to consider the wider consequences of shared decision-making is reinforced by increasing acknowledgement that achieving sustainable change in healthcare delivery systems will require attention to “organizational fields” [44] and interventions that address multiple levels of delivery systems [44, 45].

#### Healthcare system level consequences

By system, we refer to the policies and incentives that guide the work of organizations. Predicted effects at the system level are related to the power of norms and routines—in other words, “how things are normally done,” especially if these are visible, monitored, and rewarded. The proximal effect of embedding collaborative deliberation in a normative set of operational delivery processes is the influence it might have on setting the expectations for new recruits, having to learn shared decision-making, and how to use patient decision aids. Setting such norms reinforces the predicted distal and distant effects, where greater caution and skepticism may become the expected organizational cultures in healthcare settings. This new norm would contrast with settings where the early adoption of innovative treatment is routinely viewed as positive, as a way to be at the leading edge, with little consideration of a benefit-cost consideration. Greater emphasis on supporting patients to self-manage long long-term conditions, and have realistic expectations, may improve cost-effectiveness. Systems that build, sustain, and maintain such norms are likely to have significantly lower resource utilization and reductions in malpractice costs than those that resist collaboration with patients and continue frequent contacts to generate income.

This section describes many possible consequences about which little research evidence is available. To illustrate some potential ways forward, we have outlined a selection of possible research questions; see Table 2.

**Table 2:** Examples of emerging research questions

Proximal consequences	
•	Does the preferred patient role in decision-making lead to different outcomes?
•	What characteristics of patients, process, clinicians, settings and decisions moderate this relationship?
•	Is there sufficient clarity about proximal outcomes and how they are measured?
•	Do we have robust concepts as the basis for measuring decision *process*, decision *outcomes* (confidence, conflict, regret etc.), and to what degree are these mediators for distal outcomes such as treatment choice, adherence to chosen treatment, and other patient determined and patient-reported outcomes.
•	Models could be proposed and evaluated in an effort to elucidate the mediation path from a shared decision making process to a selected set of consequences.
Distal topics	
•	Do people who participate in shared decision making prior to an invasive procedure experience less distress in response to treatment side effects or adverse events than those who did not participate in shared decision making?
•	Is the distress mediated by more realistic expectations resulting from the shared decision making?
Distant topics	
•	How would resource use be affected by the implementation of shared decision making in different types of healthcare delivery settings?
•	How would implementation of shared decision making prior to specific procedures affect rates of malpractice investigations concerning such procedures?
•	How might these effects vary by type of healthcare delivery setting?

## Synthesis

By proposing a table that considers three points across time and four levels of analysis, and some potential interventions, we are able to highlight a range of potential consequences, positive and negative, that require more investigation; some are hypothesized consequences that have not been explicitly articulated previously.

At the individual level, we propose that the accumulated effects of repeated exposure to collaboration and deliberation would modify relationships with healthcare professionals and institutions. Patients, their relatives, and health professionals would be more likely to embrace caution, more adherent to options that were deliberately chosen, and, in due course, those changes could lead to different decisions about how to access and use healthcare interventions and resources.

At interactional levels, we postulate that collaborative deliberation would lead to changes at both dyadic and team levels, where peer pressure and new social norms might reinforce the establishment of deliberative decision making. At organizational levels, if incentives were aligned to support collaborative deliberation, the accumulated effects of this approach might lead to differences in utilization patterns and quality measures. Measures of patient-centered care, satisfaction, and care quality might show improvements and the burden of complaints and litigation could be reduced. If scaled across multiple organizations to healthcare system level, positive strategic and long-term dividends might result.

We based our analysis on existing reviews, attempting to stimulate thinking about more durable consequences of shared decision-making. Many have reported challenges with implementing shared decision-making and patient decision support tools, which suggests that those who manage and provide leadership for delivery systems are not yet convinced that benefits outweigh the investments required. We intentionally approached the framework from more than one perspective, consistent with recent efforts to introduce a multilevel analysis to healthcare delivery problems [30], and make it more explicit that shared decision-making might have a broader and deeper impact than has been articulated to date. We developed a generic framework so that it can be applied to healthcare delivery systems irrespective of how they might be funded, although as we note some of the consequences might be considered negative for a business model based on contact frequency or high volume procedures.

This work is provisional, being based on a number of hypotheses. Our intention is to stimulate debate as well as to generate new research questions, some of which we have outlined, in the hope that others will develop methods to study the relationships. We assume that these consequences might occur when shared decision making is consistently accomplished, avoiding known implementation challenges. We recognize that achieving such a consistent process will be difficult.

We focus on the research implications, which might provide additional information to guide healthcare delivery system investments in practice improvement. We acknowledge that existing systems do not currently accomplish shared decision-making so this assumption is not a valid starting position for researchers; research is further hampered by the need to find consensus on definitions and measures before findings can be aggregated effectively and create an evidence base considering the full range of consequences of shared decision-making.

## Research ideas

To address these issues, we propose a range of research ideas:

- Recruit and study health systems that are willing to invest in shared decision-making, at clinician, clinical team, managerial, and system levels. Ensure fidelity by measuring interactional processes at team levels. Baseline measures of team functioning, staff turnover, intervention rates, and other quality metrics would be available for comparison using time series analyses. Although some of the postulated longer term consequences might take a number of years, distal consequences might be evident sooner, such as patient-centered metrics, complaint levels, staff turnover, and team performance levels.
- Address questions at the level of teams in organizations, recognizing that high functioning teams might achieve fidelity in accomplishing new processes rapidly, and therefore exhibit the proposed consequences in less time. A range of experimental or observational designs could be used, comparing teams at different levels of motivation and performance.
- As noted, selecting healthcare systems already committed to higher quality at lower cost will be critical. Fortunately, multiple examples of such organizations are emerging as healthcare systems strive to become more cost-effective. In the USA, health reform efforts have introduced the concept of Accountable Care Organizations that are not based on fee-for-service payment models. These systems would be good settings for future evaluations. Healthcare providers in single-payer systems are also well-placed to test the effect of consistently accomplished shared decision making, provided they address the profit-driven influences of payer and provider separation. The implementation challenge is to ensure that the organizational governance and reward system is aligned with delivering consistent levels of collaborative deliberation at the front line rather than by the volume of work achieved. Research that monitors the alignment or otherwise of incentives, from the board room, to clinical management, that directly or indirectly influence front line clinicians would help illuminate the reported tensions felt by the clinical workforce. Research to compare different incentive frameworks, intrinsic and extrinsic, would be helpful.

Real-world evaluations will be expensive. Another approach would be to build models that could forecast longer term consequences, using existing evidence where available. For example, it is entirely possible that small changes in the use of diagnostic and screening tests would reduce the escalator effect of finding hitherto quiescent abnormalities that lead to further investigation and cascading intervention. Modeling the degree to which collaborative deliberation might act as a brake on overdiagnosis, overtreatment, and iatrogenic harm could help understand which changes in practice might deliver the most benefit. Further, modeling allows the comparison of competing hypotheses, and assessment of long-term relationships, without the collection of new data. Existing data from other domains, such as patient-reported outcomes from early breast cancer treatment decisions, could be used to create models and from which sensitivity analyses can guide future data collection.

## Conclusions

Shared decision-making has been welcomed by policy makers world wide—it resonates and supports the ethical imperative of respect for patient autonomy and engagement [46]. Yet, as we hope this article shows, the potential enduring benefits and unintended consequences of consistently accomplished collaboration and deliberation have not been sufficiently laid out and, therefore, not investigated.
